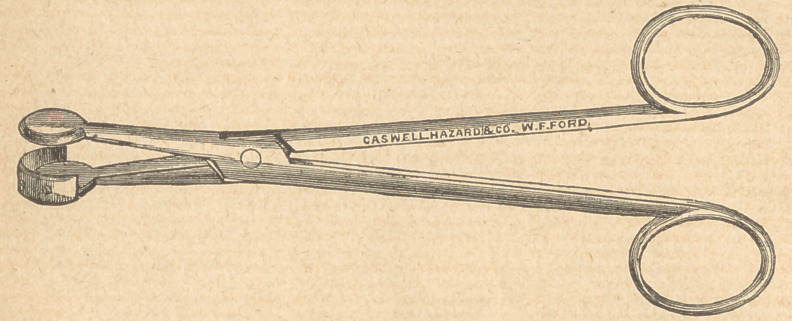# Abbott’s Scissors

**Published:** 1883-11

**Authors:** 


Under this head we will undertake, either personally or by some one of our associates, to examine and report upon such new inventions appliances and materials as may be submitted to us. Articles will be received and returned at the expense of the owners, but full directions for such return must accompany them. They will be carefully used, but we cannot be responsible for any possible loss or damage.

### ABBOTT'S SCISSORS.

The above cut represents an instrument devised by Prof. Frank Abbott, for separating the gum on erupting teeth, more especially the third molars. Every practicing dentist is aware of the extreme pain and annoyance too frequently caused by the involvment of wisdom teeth in the soft tissues about them, the inflammation sometimes extending into the pharynx and to the tonsillary and parotid glands. Simple lancing of the hypertrophied tissue is quite insufficient. There is a necessity for the removal of some portions, and for this the gum lancet is inadequate, and the operation is extremely difficult when the curved scissors are employed. It is in such cases as these that the "Abbott Scissors" will be found very useful. The cut is quite sufficient to explain the manner of their application.

## Figures and Tables

**Figure f1:**